# The psychosocial impact of pancreatic cancer on caregivers: a scoping review

**DOI:** 10.1186/s12885-025-13891-w

**Published:** 2025-03-20

**Authors:** Tara Anderson, Gary Mitchell, Gillian Prue, Susan McLaughlin, Lisa Graham-Wisener

**Affiliations:** 1 School of Nursing and Midwifery, Queen’s University Belfast, Belfast, UK; 2 Northern Ireland Pancreatic Cancer, Belfast, UK; 3 School of Psychology, Queen’s University Belfast, Belfast, UK

**Keywords:** Pancreatic Cancer, Caregivers, Family, Psychosocial, Supportive care needs, Wellbeing, Quality of life

## Abstract

**Background:**

Family caregivers are essential members of the care team of someone with pancreatic cancer, supporting their physical and psychological needs. Caregivers are often unprepared for this which may cause substantial psychosocial impact. This may be exacerbated by the short life-expectancy and rapid deterioration associated with pancreatic cancer. A scoping review was conducted to identify, from the existing literature, what is currently known about the psychosocial impact of pancreatic cancer on caregivers across the disease trajectory.

**Methods:**

A Joanna Briggs Institute (JBI) mixed methods scoping review was conducted across four databases (CINAHL, EMBASE, MEDLINE, PsycINFO). All identified citations were uploaded to Covidence, and were screened independently by two reviewers. Data were extracted and synthesised following a deductive approach guided by ‘The Cancer Family Caregiving Experience’ model (Fletcher et al., 2012).

**Results:**

42 studies were included: 22 qualitative, 15 quantitative, 5 mixed methods. Results of the included studies were collated into the proposed constructs of Fletcher et al.’s (2012) model: primary stressors, secondary stressors, appraisal, cognitive-behavioural responses, health and wellbeing outcomes, as well as the influence of disease trajectory and contextual factors. The literature highlighted pancreatic cancer caregivers experienced stress related to caregiving activities, disruptions in their daily life and family relationships, high levels of unmet need, and poorer quality of life compared to other cancer caregivers. They were also at increased risk for various psychiatric disorders and reported a persistent lack of support which exacerbated the psychosocial impact.

**Conclusions:**

Pancreatic cancer caregivers experience negative psychosocial impacts, exacerbated by the disease’s trajectory. Feelings of a lack of support were reflected throughout the included literature and emphasise the need for future research into how pancreatic cancer caregivers may be best supported, and sign-posted to existing support, to minimise the substantial psychosocial impact they may experience.

**Supplementary Information:**

The online version contains supplementary material available at 10.1186/s12885-025-13891-w.

## Background

Pancreatic cancer is a challenging and aggressive disease with high mortality rate [1–3]. With 5-year survival rates estimated at 9% [4], pancreatic cancer represents a serious public health concern worldwide. The majority of patients present symptomatic at an advanced stage [1, 3] and are not eligible for potentially curative treatment [2]. Patients often experience high psychological and physical symptom burden, which can have a sustained impact on their quality of life (QoL) [5–7]. For example, pancreatic cancer is associated with a high prevalence of cancer-related depression and anxiety compared to other cancers [8–10], as well as high levels of pain [11, 12] and specific gastrointestinal problems such as malnutrition resulting from pancreatic exocrine insufficiency [13, 14].

Caregivers, such as family members or friends, often take on a role of supporting patients’ physical and psychological needs, becoming an essential member of a cancer patient’s care team [15, 16]. Caregivers tend to be unprepared for this role and report negative impacts on their own health [17], high psychological burden [18], distress [19], and unmet needs [20, 21]. Within the context of pancreatic cancer, specifically, the short life-expectancy and rapid deterioration associated with the disease may exacerbate these experiences [22].

Fletcher et al. (2012) have synthesised previous research regarding the cancer caregiving experience into a conceptual model, ‘The Cancer Family Caregiving Experience’, consisting of three main elements: the stress process, contextual factors, and the cancer trajectory [23]. The included stress process model is based upon the Transactional Model of Stress and Coping [24] and its previous applications to the cancer caregiver population [25]. The expanded stress process model consists of five broad constructs: primary stressors (e.g. patient illness-related factors), secondary stressors (e.g. caregiving role, relationships, financial stress), appraisal (e.g. caregiver burden and unmet need), cognitive behavioural responses (e.g. avoidance, acceptance, developing caregiving skill), and health and wellbeing outcomes (e.g. anxiety, QoL) [23]. The term psychosocial impact will be used as an umbrella term to refer to these outcomes as this incorporates the social and psychological aspects of an individual’s life which are influenced by such stressors as well as the individual’s appraisal and response [26, 27].

This model highlights the importance of considering the impact of both contextual factors such as the economic and cultural environment as well as the stage in the disease trajectory on caregiver’s experiences. As the majority of pancreatic cancer cases are diagnosed at an advanced stage [1, 3], the disease trajectory may be accelerated which in turn may influence the stress process experienced by caregivers. Therefore, pancreatic cancer presents unique challenges for caregivers who may be quickly thrust into a challenging role, taking care of patients with high symptom burden and rapid deterioration [28, 29] while they are also coping with the news of an often-incurable diagnosis. This conceptual model provides a framework for understanding the complexities of family caregiving in cancer and will enable the synthesis and organisation of existing literature regarding the experiences of pancreatic cancer caregivers.

Two recent systematic reviews have been conducted regarding the experiences of pancreatic cancer caregivers. One of these focuses solely on QoL as an outcome measure for both pancreatic cancer patients and caregivers and as caregiver experiences were not the main focus of this review, this is not discussed extensively [5]. The second systematic review explored pancreatic cancer caregivers’ burden, unmet needs, and QoL, across studies which focused solely on pancreatic cancer [22]. The present review aims to add to this literature by incorporating studies which include a subset of pancreatic cancer caregivers. The addition of such data may provide further insights into their experiences, and there exists a need to chart this broader range of evidence. An initial search of the literature also identified at least eight potentially relevant studies published since the completion of the most recent review suggesting a recent increase in research related to pancreatic cancer caregivers. Finally, incorporation of ‘The Cancer Family Caregiving Experience’ model will provide a theoretical foundation to explore the breadth of existing research in this area and enhance the present review’s ability to interpret diverse findings to obtain a more comprehensive understanding of, and greater insights into, the psychosocial impact of pancreatic cancer on caregivers.

To chart the existing evidence related to the psychosocial impact of pancreatic cancer on caregivers, the present scoping review (ScR) sought to explore psychosocial outcomes, as well as contextual and disease trajectory factors, in line with ‘The Cancer Family Caregiving Experience’ model [23]. The ScR method has been chosen to facilitate the comprehensive overview of existing evidence for exploratory purposes [30]. To do so, this ScR aimed to identify from the existing literature what is currently known about the psychosocial impact of pancreatic cancer on caregivers across the disease trajectory via the following objectives:

1. To examine the psychosocial impact of pancreatic cancer on caregivers, in line with ‘The Cancer Family Caregiving Experience’ conceptual model.
2. To synthesise the evidence on the psychosocial impact of pancreatic cancer on informal caregivers across the disease trajectory, from diagnosis to survivorship or bereavement.
3. To identify any gaps in existing research regarding the psychosocial impact of pancreatic cancer on informal caregivers across the disease trajectory.

## Methods

### Protocol and registration

This ScR was conducted in line with the Joanna Briggs Institute (JBI) methodology for ScRs [31] and reported in accordance with the Preferred Reporting Items for Systematic Reviews and Meta-analyses extension for Scoping Reviews (PRISMA-ScR) checklist [32]. A protocol for this ScR was registered prospectively on Open Science Framework on 29 February 2024 (registration DOI: 10.17605/OSF.IO/NEKZ4).

### Eligibility criteria

#### Types of sources

Analytical observational studies including prospective and retrospective cohort studies, case-control studies and analytical cross-sectional studies were considered for inclusion. This review also considered descriptive observational study designs including case series, individual case reports and descriptive cross-sectional studies for inclusion. Studies were also considered that focused on qualitative data including phenomenology, grounded theory, ethnography, and qualitative description. Systematic reviews identified were searched for relevant studies found in their reference sections that may not have been found in the database searches. Studies published in English only were included due to a lack of language diversity amongst the researchers. No date restrictions were applied, and grey literature was not included. Further eligibility criteria was developed using the Population, Concept, and Context (PCC) framework as recommended by JBI [31].

##### Population

The population of interest were current or bereaved caregivers of patients with pancreatic cancer. This included spouses, patient’s adult children, family members, friends, and neighbours. No restrictions were placed on gender, age, or ethnicity. Studies were considered for inclusion if at least some participants were pancreatic cancer caregivers, for example those which included both pancreatic cancer patients and caregivers, or those which included caregivers of patients with other cancers. However, such studies were only included when at least 50% of the participant population were pancreatic cancer caregivers or their data could be extracted.

##### Concept

Studies which reported on the psychosocial impact of pancreatic cancer on caregivers across the disease trajectory were considered for inclusion. This included both quantitative and qualitative research which reported on outcomes such as quality of life (QoL), caregiver burden, psychological wellbeing, and supportive care needs. No restrictions were placed on the stage of the pancreatic cancer trajectory.

##### Context

Studies conducted across all settings (e.g. hospitals, hospice, primary care, community-based) were included in the review. No restrictions were placed on geographic location.

### Search strategy

An initial limited search of MEDLINE, PsycINFO, EMBASE, and CINAHL was undertaken to identify studies on the topic. The text words contained in the titles and abstracts of relevant studies, and the index terms used to describe the studies were used to develop a full search strategy for MEDLINE (Supplementary Material 1). This search strategy, including all identified keywords and index terms, was adapted for each included database and received input from a subject librarian. To identify potentially relevant studies, the following bibliographic databases were searched on 4th March 2024: MEDLINE, PsycINFO, EMBASE, and CINAHL. The reference list and citations of all included sources of evidence were also screened for additional studies. This search was repeated on 24th November 2024 to screen for any newly published studies.

### Selection of sources of evidence

Following the search, all identified citations were collated and uploaded into Covidence (https://www.covidence.org/), a screening and data extraction tool for streamlining the production of reviews. Following the removal of duplicates, TA and GM each independently carried out full screening of the title and abstracts, with the same procedure used for full text screening. And disagreements throughout the screening process were resolved following a discussion between TA and GM.

### Data charting

Charting the data was carried out in Covidence, guided by the ‘JBI template source for evidence details, characteristics and results extraction instrument’ [33]. TA conducted data extraction from the articles included in the scoping review, and this was checked for accuracy by GM independently. The data extracted included specific details about the author, year, setting, participants, study methods, outcomes, and key findings.

**Fig. 1:**
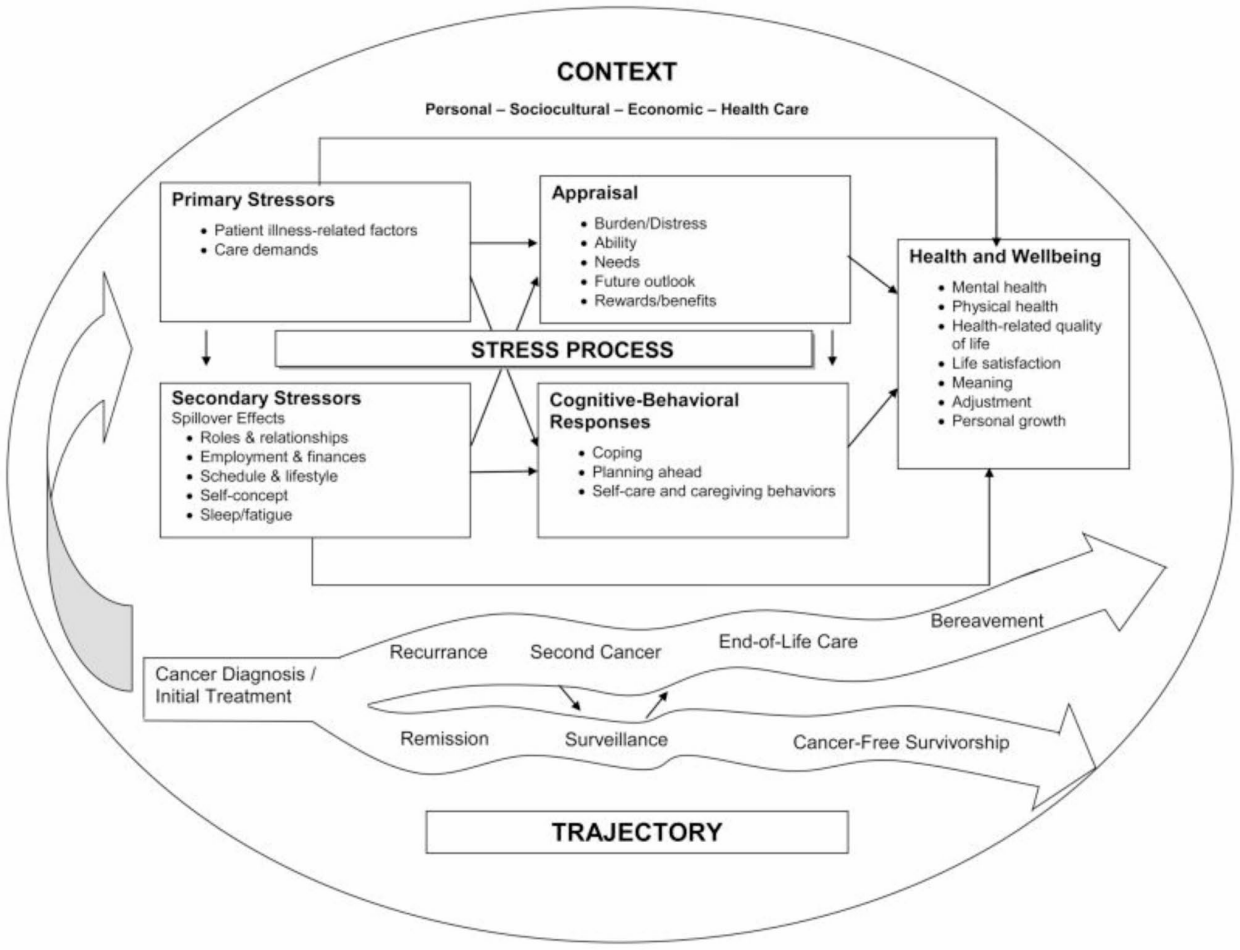
The cancer family caregiving experience conceptual model [23]

As recommended by JBI ScR guidance, a basic qualitative content analysis approach was utilised to analyse and present the results of the included studies [31, 34]. This followed three phases: (1) preparation, (2) organising, (3) reporting, in line with recommendations [34, 35]. A deductive approach was taken to extract data according to ‘The Cancer Family Caregiving Experience’ conceptual model [23] (Fig. 1). Results, therefore, from the charting process were collated into the constructs proposed within this model: primary stressors, secondary stressors, cognitive appraisal, cognitive-behavioural responses, and health and wellbeing, as well as the influence of contextual and disease trajectory factors.

## Results

**Fig. 2:**
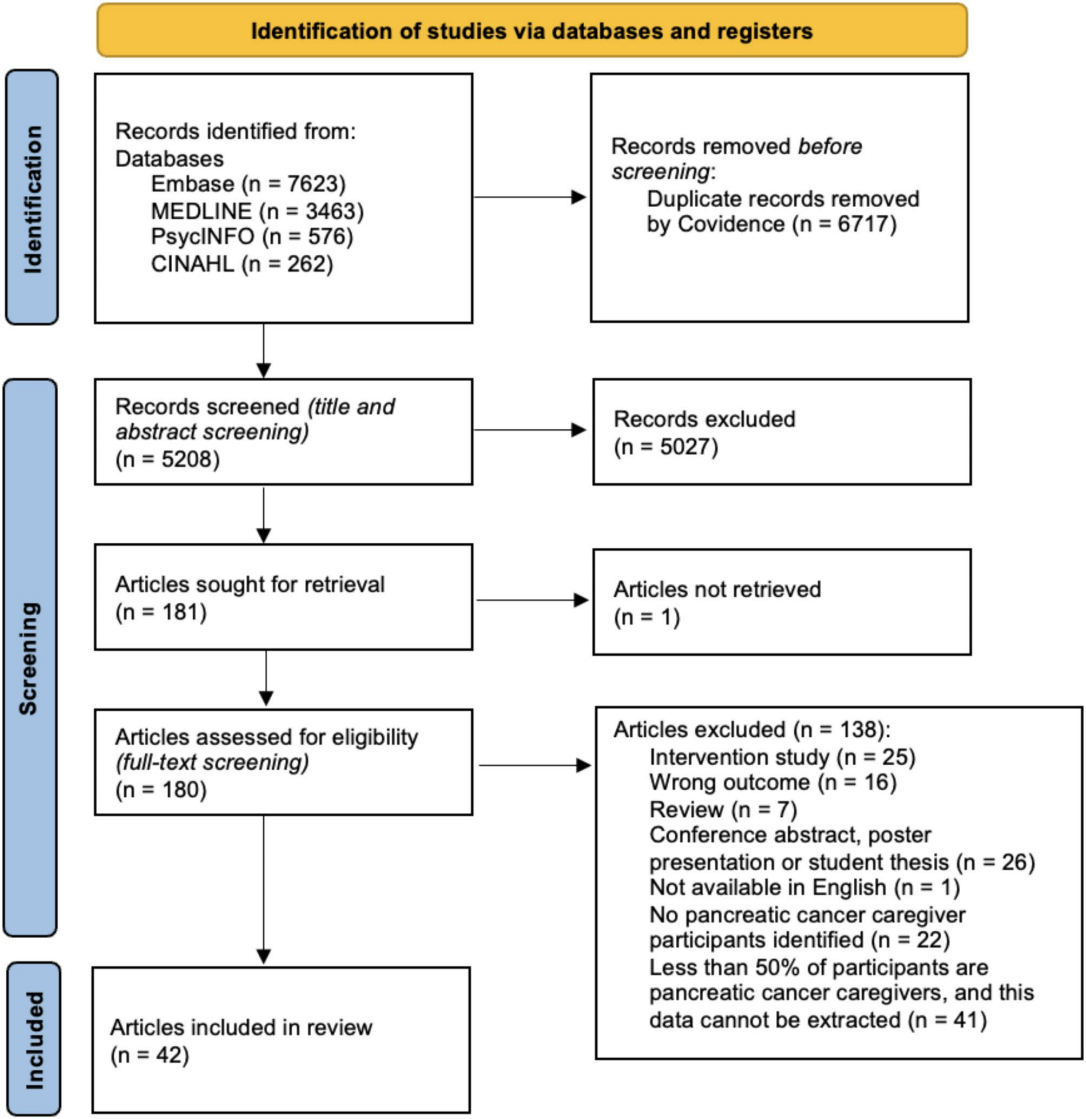
PRISMA flow diagram

The search strategy yielded 11,924 results. This was reduced to 5,208 after duplicates were removed. Following title and abstract screening, 5,027 articles were excluded as they did not meet the inclusion criteria while one could not be found. Reasons for exclusion at this stage included articles which reported on different populations (e.g. other cancer caregivers, cancer patients), and different outcomes (e.g. familial risk of cancer, clinical trials). After the full-text review stage, 42 articles were deemed eligible and included in the final review. A PRISMA-ScR flowchart has been provided to summarise the study identification process in Fig. 2.

Three of the included studies from the USA report on the same dataset of pancreatic cancer caregivers and the linked clinical data of the patient [36–38]. In addition, two Australian studies included report on data from the Queensland Pancreatic cancer Study– Quality of Life, a longitudinal, repeated-measures study of patient-caregiver dyads after a pancreatic cancer diagnosis [39, 40]. Therefore, 42 articles are included which report the data of 39 studies.

### Characteristics of included studies

The 42 studies were published between 2001 and 2024 (Supplementary Material 1). The majority were published within the past five years (*n* = 24), 14 of which were published in 2023. 22 were qualitative [41–62], 15 quantitative [36–40, 63–72], and five used mixed methods [73–77]. Included studies were conducted in a range of countries, most often the USA (*n* = 16) [36–38, 41, 51, 52, 54, 56, 58, 65, 70–72, 75–77], followed by Australia (*n* = 6) [39, 40, 45, 49, 50, 57] and China (*n* = 5) [61, 62, 67–69]. Other countries included Denmark (*n* = 3) [44, 63, 64], The Netherlands (*n* = 3) [43, 46, 59], Sweden (*n* = 2) [47, 74], Japan (*n* = 2) [48, 53], and one study each from France [73], Germany [60], Greece [55], and the UK [42]. One study included participants from both Denmark and Sweden [66].

Sample sizes of included studies ranged from one [41, 48] to 5,774 pancreatic cancer caregivers [63]. For two studies, however, it was not clear how many participants were pancreatic cancer caregivers; a qualitative study of 30 family caregivers [51] and a quantitative study of 546,321 spouses of patients with cancer [66] although only findings related to pancreatic cancer caregivers were extracted. Exact sample numbers are not available for all included studies, but it is evident the majority of caregivers were female and the partner of the pancreatic cancer patient. Other participants included the patient’s children, parent, sibling, or friend. Bereaved relatives were included in some studies (*n* = 13) [45, 48, 49, 52–57, 59, 63, 65, 74], but the majority were current caregivers. Eight studies reported on only pancreatic cancer caregivers [48, 54–56, 63, 69, 76, 77], the majority included caregivers of other cancers and/or patients (only data regarding pancreatic cancer caregivers was extracted from these).

Of the qualitative studies, six utilised thematic analysis [42, 47, 54, 55, 60, 62], one used reflexive thematic analysis [49], four used thematic content analysis [45, 46, 57, 59], three used qualitative content analysis [44, 58, 61]. Other analyses included framework analysis following a grounded theory approach [43], a Heideggerian hermeneutic phenomenological approach [50], and constant comparative methods [51, 52]. Finally, four were case reports [41, 48, 53, 56].

A range of measures were utilised within the included quantitative studies. Studies which examined psychological wellbeing largely focused on anxiety and depression utilising measures such as the Hospital Anxiety and Depression Scale (HADS) [39, 40, 67, 69]. Other measures included the Supportive Care Needs Survey for Partners and Carers (SCNS-P&C) [39], as well as measures of coping style [64, 67, 68], and caregiver burden [69, 70].

The majority of the included mixed-methods studies utilised a survey and interviews. Measures included the Caregiver Reaction Assessment (CRA) and SCNS-P&C [73] and measures of QoL [76, 77] while thematic analysis was commonly used to analyse the qualitative data [73, 75, 77].

### Psychosocial impact of pancreatic cancer on caregivers

Results are presented in line with the three main components of ‘The Cancer Family Caregiving Experience’ model; the stress process, disease trajectory, contextual factors [23].

#### The stress process

Results of the included studies were categorised and collated into the proposed constructs of the stress process. Table 1 provides an overview of the categories identified within each construct.

**Table 1:** Categories identified within constructs of ‘the Cancer family caregiving experience’ model’s proposed stress process [23]

Primary Stressors	Secondary Stressors	Appraisal	Cognitive-Behavioural Responses	Health and Wellbeing
Caregiving activities	Changing roles and relationships	Unmet needs	Coping processes	Anxiety and depression
Patient’s psychological wellbeing	Employment and financial impact	Perceived caregiver burden	Spirituality	Quality of life
Patient’s gastrointestinal symptoms		Future Outlook	Support from others	Other impacts on health and wellbeing
		Perceived rewards and benefits	Development of caregiving knowledge and skill	

##### Primary stressors

Patient illness-related factors and the resulting caregiving demands are considered primary stressors [23]. This includes the patients’ prognosis and symptoms which initiate the stress process for the caregiver as they help to manage these factors [25]. Primary stressors were evident across many of the included studies (*n* = 21).

Caregiving activities

Firstly, caregiving activities were referenced across 17 of the included studies; 10 qualitative [43–47, 49, 54–56, 60], three quantitative [38, 65, 70], and four mixed methods [73, 75–77]. These included assistance with transportation, housework, self-care, exercise, diet, and monitoring symptoms [37, 46, 49, 54, 73, 77].

Other tasks included talking to doctors, arranging and attending medical appointments, and updating other family and friends about the patient’s status [65]. Pancreatic cancer caregivers reported visiting an average of 4.7 doctors (e.g. general practitioner, gastroenterologist, medical oncologist) and co-ordinating care between these doctors [65]. Medical assistance such as care of surgical drains, wounds, and medication management was described [44, 55]. Caregivers typically engaged in such activities seven days a week for seven to nine hours a day [75, 76]. The number of daily caregiving activities was found to predict caregiver distress [70]. Qualitative research provides an insight into this as a female caregiver whose husband had died from pancreatic cancer in the USA commented: “To me, it just never stopped. It wasn’t the care, it was the whole commitment. It never went away” [56].

Caregivers felt they were largely invisible to healthcare professionals but expected to undertake many practical and logistical tasks related to the patient’s disease, treatment, care and rehabilitation while having to manage housekeeping, work, and support the patient psychologically [44]. Management of medications, dosage and administration were described as overwhelming [56]. Caregivers reported difficulties with tasks which resembled nursing care as shown in a qualitative study conducted in Greece in which a caregiver to her husband commented: “I did things I never thought I’d do before this thing happened. Like the injections in the abdomen… it’s not an easy thing for a person who is not used to or trained in such things” [55].

Caregivers felt they were one person trying to fulfil many different roles (e.g. husband/wife, nurse, secretary and pharmacist) due to the range of responsibilities they took on which were “mentally draining and extremely frustrating” [60]. A female caregiver in a qualitative study conducted in the USA commented “Where do we begin, we need help” [60]. Caregivers acknowledged that they were important partners within the decision-making process, for example regarding treatment, but felt they did not know enough to contribute [43].

The lack of information received regarding symptom management, postoperative complications and discharge planning lead to feeling unsupported [45, 47]. One caregiver, in a qualitative study conducted in Denmark, felt unsupported in providing home care “But what if something happens. What if he starts screaming with pain at two in the morning or if the drain falls out? What precisely do I do?” [44]. Caregivers felt helpless in their ability to manage treatment side effects, as a male caregiver in a qualitative USA study explained: “Well, I am helpless. I can give her emotional support but I can’t cure the cancer, I can’t make her not nauseous, I can’t make her go to the bathroom, I can’t make her eat normally… so it’s very frustrating because there’s so many things out of my control” [60].

Impact of the patient’s psychological wellbeing

The psychological wellbeing of the patient was also found to impact caregiver distress and supportive care needs across three included quantitative studies [39, 40, 70]. People with pancreatic cancer may experience substantial psychological burden which impacts on the wellbeing of their caregivers, for example, patient’s distress was significantly correlated with caregiver’s distress, perceived stress, anxiety, depression, and burden [40, 70]. One mixed-methods study described this as reciprocal suffering where caregivers were “up when the patient is up and down when the patient is down” [76]. Additionally, caregivers who reported more unmet supportive care needs were more likely to be those caring for a patient with anxiety and/or depression compared to those who did not report any moderate-to-high needs [39].

Patient’s Gastrointestinal symptoms

Seven studies explicitly highlighted the negative impact of the gastrointestinal symptoms associated with pancreatic cancer on the caregiver which were commonly experienced across all stages of the cancer journey; five qualitative [45, 49, 51, 58, 60], one quantitative [38], and one mixed methods [75]. Stress surrounding the patient’s dietary needs and restrictions were identified [38]. Management of symptoms associated with the gut and diet were reported as issues which caregivers felt contributed to significant distress [45, 58, 75]. A lack of support was discussed in relation to managing these symptoms [49] which participants expressed a lack of access to dieticians as a potential reason for [45].

In one study, participants expressed frustration that they had not been provided with basic information about pancreatic exocrine insufficiency and felt physicians were reluctant to prescribe pancreatic enzyme substitution therapy to address this [45]. Participants in this study felt if they had had more information and received this treatment earlier this may have relieved unnecessary suffering and discomfort. For bereaved caregivers, this issue stood out as having increased distress and contributed to feelings of unresolved grief as they felt if these symptoms had been addressed it would have made a significant difference to the patient’s experience through to the end of their life. A bereaved male caregiver in an Australian study described: “Well the pancreatic enzyme made such a huge difference. If we’d only known, we could got that earlier. That would have been great” “We only got it about a week or 10 days [before she died] and it would have been better if we’d had them a month earlier” [45].

##### Secondary stressors

Secondary stressors are those which arise from aspects of caregiving as a result of the primary stressors, including changes in roles and family structures, financial and employment stress [23]. Across the literature included in this review, a number of these factors were discussed in 19 included studies.

Changing roles and relationships

Caregivers described changes in their roles and family relationships across ten studies which utilised qualitative methods [45, 51, 54, 56, 58, 60, 73, 74, 76, 77]. Caregivers struggled with balancing multiple roles including caring for the patient, themselves, children, spouses, and jobs [60, 73, 76, 77]. One caregiver, in a USA study, explained “I mean everything has changed to kind of revolve around [the patient’s] treatment. So, my [other family members] has definitely gotten a lot less attention since all this happened” while another commented “…little chores around the house that kind of were his thing to take care of, I’m having to do all of that and you know when the kids call and they need something, it falls on me to make sure that… they get the help they need…” [77]. A caregiver to his father, in a USA study, described a need to manage aspects of his own life “I had to take care of my children. Try and straighten out my financial matters and household and… have to work of course” [54]. Spousal caregivers discussed attempting to maintain their ‘husband-wife relationship’ without this becoming a ‘patient-nurse relationship’ [60]. One caregiver whose husband had advanced pancreatic cancer commented “He’s not even with me… it’s like cancer is married to him now and I’m not. So, the cancer is with him all the time and I’m [alone] in the room” [58].

Rather than changes in family dynamics, caregivers in one study reported that the experience heightened already established dynamics [54]. As one pancreatic cancer patient’s son explained, in a USA study: “How families deal with things is how they’re going to deal with this. Kind of almost predetermined in their relationship than it is something that’s pancreatic specific, I guess” [54]. Similarly, a wife commented “I knew I would take care of him myself because we had always done things together. We worked together for 30 years… we did everything together since I was 18 years old… There was never a question that I wouldn’t continue to be [with him] during this period” [56].

While some families were brought closer together by the diagnosis, others were divided [54]. This was sometimes accepted, as a sibling of a pancreatic cancer patient in a USA study commented: “we are all individuals, and I think we kind of reacted as individuals” [54]. While other times caregivers reported resentment due to this distance between family members, as a caregiver to a parent in the same study described: “I feel like… there should be more of the share of the responsibility instead of myself running the whole show and dealing with it, emotionally and financially. So, I feel there is a great sense of resentment.” [54]. The impact of caregiving on family dynamics was highlighted in another study as when families spent more time together during the illness period this exacerbated pre-existing negative family dynamics and issues [76].

Employment and financial impact

The impact of caregiving on employment and finances was evident across seven studies; four quantitative [36, 38, 65, 69] and three mixed methods [73, 76, 77]. Quantitative data identified caregivers who were in salaried employment were more likely to report emotional and financial difficulty than those who were not working [36]. In a USA study, some working caregivers felt a pressure to remain at their job to retain health insurance [38]. In a study of 213 caregivers, 40% reported having to leave employment to care for their loved one [65]. While a smaller study of 28 caregivers of those with localised pancreatic cancer found similar patterns, with 47% of participants reporting financial challenges caused by decreased working hours or having to leave their job [77].

Similar findings were expressed qualitatively as caregivers expressed that both their working hours and productivity while at work suffered [76, 77]. The financial impact became apparent in comments such as: “I was working four or 5 days a week just for extra money before all this happened and now, I can’t do that anymore. So that’s kind of hurting a little.” [77]. Another caregiver discussed how he was closing his law practice to be able to spend more time with his wife who had pancreatic cancer: “I’ve missed a lot of work and I’m actually switching jobs because of this to make it easier for me to be able to do things with her… I was self-employed… I can’t do that now so I’m literally closing my practice” [77]. This same caregiver reasoned that even if he had kept on this job, he wouldn’t have been able to focus on it due to concern for his wife: “I wouldn’t be able to really mentally be in my job, because I’d be there worrying” [77].

##### Appraisal

Caregiving appraisal encompasses all cognitive and affective appraisals and reappraisals of potential stressors as well as the caregiver’s beliefs regarding their ability to cope with those stressors [78]. Within ‘The Cancer Family Caregiving Experience’ model, caregiving appraisal is conceptualised as distress appraisal (e.g. caregiver burden), evaluation of needs, future outlook, as well as rewards and benefits [23]. These factors were demonstrated across the majority of studies (*n* = 30).

Unmet needs

Thirteen studies found evidence of unmet caregiver needs [38, 39, 44–46, 55, 56, 60, 65, 73, 75–77]. Two studies explicitly measured unmet supportive needs utilising the SCNS-P&C [39, 73]. Although sample sizes differed greatly, a high prevalence of unmet needs were found in both studies. One study surveyed seven French pancreatic cancer caregivers, with 93.7% reporting unmet supportive care needs [73]. While the other found that 63% of 84 caregivers in Australia reported at least one moderate-to-high unmet need [39]. The highest number of unmet needs were found in the healthcare services and information domain of the SCNS-P&C [39, 73]. This is supported within the qualitative literature included where information needs were highlighted across several studies. This included a lack of information about treatment options, prognosis, symptom and medication management [46, 47, 49, 56, 77]. When information was provided, some caregivers reported it did not meet their needs due to volume, timing, and medical jargon [46].

Emotional and psychological needs were also identified, ranking as the second highest scoring domain of unmet needs in one quantitative study [73]. Another study found caregivers of individuals with advanced pancreatic cancer reported needing someone to address their feelings and acknowledge the physical, emotional, social, and spiritual impacts associated with illness, loss, and the anticipated death of someone they loved [76]. Caregivers reported feeling that although the patient had them for emotional support, there was no support available to them as one commented, in a qualitative study conducted in Australia: “My husband being the patient, obviously they were offering the counselling to him. If I was the doctor, I would have said, okay, you’ve got your wife, but I would have asked me the question, then, how are you coping with all this, you know?” [49]. Similarly, another female caregiver in a USA study commented: “Even when you go in and see [the doctor] or any of the other providers, it’s about them [the patients]. Nobody says how are you doing?” [60].

Despite the evident unmet caregiver needs, caregivers often focused on the patient’s preferences and needs over their own [55, 75]. One study found the top three issues of caregivers all related to the patient; the patient’s QoL, extending the patient’s life, and managing the patient’s symptoms [65]. The authors of a qualitative study noted that when asked about unmet needs, caregivers often could not readily identify their own needs despite many coming across throughout their interviews [76]. In addition, caregivers were found to recognise the need for self-care but felt selfish and guilty to take time away from the patient and so “put ourselves on the backburner” [60].

Perceived caregiver burden

The demands and stressors of caregiving may be evaluated as caregiver burden which was evidenced across four included studies; two quantitative [64, 69] and two qualitative [55, 59]. In one study, the majority of participants reported mild perceptions of caregiving burden, in this study greater benefit finding and greater self-efficacy were associated with lower perceived care burden [69]. Another found high perceived caregiving burden in a small number of caregivers at both baseline and nine-month follow-up [64]. Qualitative literature provided an insight into caregiver burden, for example, participants described the responsibility to make ethically complex decisions about treatment psychologically burdensome [55], while others found frequent appointments and treatment schedules during chemotherapy burdensome [59].

Future outlook

Appraisal was also evident as caregivers described their worries and hopes for the future in seven included qualitative studies [44, 50, 52, 54, 55, 60, 62]. This including feelings of uncertainty and foreboding [44], fear of the future [60], and preparatory mourning [55]. In one study, caregivers became hypervigilant of symptoms and sceptical of a good prognosis, even when presented with good news from the medical team [60]. One female caregiver in a USA study presented a photograph of a dark cloud to signify the constant overshadow of doubt and worry: “No matter what kind of wonderful day you’re having, you know that these black clouds are there, and, on any day, life could change again in a minute. So, you never ever really are without feeling that” [60]. On the other hand, some caregivers were hopeful that medical advancements may lead to better outcomes, as the spousal caregiver in a study conducted in China commented: “With the advancement of science and technology, I believe that tomorrow will be better” [62].

Perceived rewards and benefits

Some evidence of benefit finding during the caregiving experience was reported in eight of the included studies; five qualitative [41, 42, 52, 59, 61], two quantitative [65, 69] and one mixed methods [76]. For example, in an online chat room, several family members shared with others the blessings for which they were thankful (e.g. a supportive friend, a helpful nurse, being able to take time off work to be with their loved one) [52]. In other studies, caregivers discussed an appreciation for spending more time with the patient [41, 59, 76]. For example, when they knew their loved one had a terminal diagnosis: “The knowledge [that the disease is terminal], that is really tough and painful… but you are able to say goodbye” [59].

In one study, only 5.2% of caregivers reported a determined or positive outlook [65]. Similarly, a qualitative study reported that the majority of caregivers felt there were no gains to caregiving although some caregivers reported that, through caregiving, they had discovered their own strength, which in turn, increased their self-efficacy [76]. Another study found caregivers appreciated recognition from those they were caring for as one caregiver commented: “My father was not very conscious, but he always smiled at me when he opened his eyes, which was actually his way of expressing his gratitude to me. All I did was worth it” [61]. In one case, the son of a pancreatic cancer patient described how his father’s outlook of feeling lucky and appreciating day-to-day life, helped him to accept the diagnosis as he commented: “[the idea of his dying] went from being something horrible to being something that’s just there. I try to see it the way he sees it: that at some age it’s what’s going to happen to everybody” [41].

##### Cognitive-Behavioural responses

Cognitive and behavioural responses are the individual’s thought processes and actions taken in response to their situation [23]. ‘The Cancer Family Caregiving Experience’ model considers coping mechanisms (e.g. acceptance, reframing, distraction, denial, planning and avoidance) as well as development of caregiver knowledge and skill as cognitive and behavioural responses. The cognitive-behavioural response of caregivers was evident across 21 studies.

Coping processes

Different coping strategies were evidenced across 11 of the included studies; six qualitative [44, 54, 55, 59, 61, 62], three quantitative [64, 67, 68], and two mixed methods [76, 77]. Quantitative data highlighted that increased dyadic coping mediated caregiver’s anxiety and depression [67] while more positive coping styles (e.g. seeking advice from family and friends and participating in recreational and sports activities) positively impacted caregiver QoL [68].

Qualitative data revealed that caregivers admitted to hiding their feelings from those around them during the illness period [62, 76]. In one study, they reported they had not been conscious of doing this at the time but that it was their natural reaction to the situation [54]. One caregiver, in a USA study, commented “I think I felt like I needed to be the strong one and just really kind of numb myself for a while and not respond for a bit” [77]. In one study, this was described as ‘keeping a stiff upper lip’ as caregivers felt they had an obligation to contain their own emotions of anxiety and fear [44]. In a study conducted in Denmark, another caregiver commented, “I know this isn’t a “good” cancer… but I stay positive and say to him: Dad you’re one of the good cases…” [44].

Some caregivers discussed acceptance of the diagnosis and their loved one’s prognosis [55, 59, 77]. In one study, 47% of caregivers reported that facing their reality via acceptance or rationalisation was the only way to move forward, as one caregiver shared, “I guess there’s no way around that. Just kind of suck it up and face it” [77]. The most common coping strategies employed in one study were maintaining hope in regard to the patient’s prognosis (73%) and focusing on the present (67%) [77]. Other coping mechanisms included humour, comparisons with others such as those who were sicker or who had died (e.g. “things could be worse”) and self-medicating (e.g. increased smoking and alcohol intake) [44, 76].

Denial and downplaying of the experience were evident in a number of studies. For example, caregivers downplayed both the severity of treatment and their emotional response “My emotions are in supporting her and what needs to be done. So, yeah it hasn’t affected me, no” [77]. Caregivers also reported choosing not to think about their situation or chose what to hear when receiving news from a healthcare professional [76].

Spirituality

Spirituality was discussed by some caregivers in four studies utilising qualitative methods, with faith in God an important aspect of coping [52, 54, 76, 77]. Caregivers reported attending church services and engaging in religious observance in private to help them cope with their situation [54]. “We have faith, we trust in God, and we are praying that God will take care of us, take care of him, and all this situation” [77].

One study analysed the spiritual issues found in postings of family members on an online pancreatic cancer chat room [52]. Promises of prayer and requests for prayer were commonly observed, sometimes this involved specifics such as praying for the shrinkage of tumours. Some posts highlighted feelings of conflict between spiritual beliefs and suffering, such as why God wanted their loved ones to suffer or was punishing them with the disease. Some posts referred to pancreatic cancer as a “monster” or “beast”. Others asked God to take their loved one so they would not suffer anymore.

Support from others

Support, or a lack of support, from others with the illness experience was evident within sixteen studies; eight qualitative [43, 44, 49, 54, 55, 57, 60, 62], four quantitative [36, 37, 40, 65], and four mixed methods [74–77].

Qualitative data provided an insight into support caregivers engaged with as they took comfort in talking to those who had a similar experience [54]. While on the other hand, some felt they could not open up to family and friends and lacked formal support [49, 54]. For example, a caregiver to their parent in a study conducted in the USA commented: “There was no support system. A support group or any group or resources out there that would assist the family” [54].

The need for increased support was evident as caregivers discussed feeling unsupported and unprepared for caregiving duties [37, 74, 75]. Caregivers reported low preparedness for caregiving [75] and experienced powerlessness and helplessness related to their perception of the patient’s suffering as well as their feelings of insufficiency [74]. Caregivers felt that healthcare professional communication in relation to caregiver understanding and support was poor [37]. In this same study, the majority of caregivers reported that they had never been asked whether they had the skills or training to care for the patient.

Only half of the caregivers in an American study reported having used a support service, while those who were aware of one were less likely to report negative emotions [65]. Some caregivers, who had found community support services themselves, reported these were useful in providing plain language information, emotional support, and signposting to support groups [49]. However, caregivers had to seek this out themselves and felt this was something their healthcare providers could have pointed them towards.

A minority of participants, in one study, sought professional therapy, and those who did, were often experiencing other hardships such as having another family member or close friend with cancer [54]. Professional support was described as helpful in sorting out and dealing with feelings [54]. In another study, accessing professional psychological help was significantly associated with subclinical levels of anxiety, as assessed by HADS [40].

Caregivers often expressed their hopes for how services may change to better support others and provided feedback on what had benefited them, as referenced within the qualitative data of seven included studies [43, 44, 55, 57, 62, 76, 77]. Recommendations included support groups and improved communication between care providers [77], and a follow-up ‘caregiver consultation’ with a healthcare provider to discuss concerns and questions [44]. In one study, caregivers expressed an appreciation for having a space to share their feelings with one commenting that their participation within that study “was a blessing” [76].

Development of caregiving knowledge and skill

Developing caregiving knowledge and skill in an attempt to cope with their experience was referenced across nine studies; seven qualitative [44, 45, 49, 54, 58, 59, 61], one quantitative [38] and one mixed methods [77]. Information seeking was highlighted as a common coping mechanism as caregivers attempted to understand the disease prognosis [44, 49, 54, 75]. Specific areas were highlighted in which caregivers felt they would have liked to have been better prepared for including management of the patient’s dietary symptoms and postoperative care [38, 45, 75]. In an Australian study, caregivers discussed a lack of information and support following discharge from hospital after surgery as one commented: “The hospital made no attempt to explain to me what I needed to do. I had to do basically a crash course in how to use the feed, how to feed her, how to look after her and I found that very difficult in the first week or so with working out exactly what I needed to do” [49].

As previously discussed as a primary stressor, dietary symptoms caused significant distress. Caregivers attempted to ease this through discussing their concerns with an oncology dietician, where available, and being more conscious of what they cooked (e.g. more vegetables and less red meat) [75] and trying to prepare meals the patient may enjoy [58, 59]. In another study, caregivers reported using the internet to source information about diet and gastrointestinal symptoms [45]. Caregivers also utilised the internet for information about the disease more generally [44, 54, 77]. The wife of a pancreatic cancer patient undergoing home-based palliative care, in a study conducted in China, discussed joining an online family help group: “Whenever I have a care problem, I ask in the group, and there is always someone who can give me advice” [61].

##### Health and wellbeing

The health and wellbeing domain of ‘The Cancer Family Caregiving Experience’ model encapsulates the mental and physical health outcomes of the stress process [23]. Outcomes such as anxiety, depression, and QoL were identified across 21 of the included studies.

Anxiety and depression

Symptoms of anxiety and depression were evident in 11 of the included quantitative studies [38–40, 63–67, 69, 70, 72] and reported in three studies utilising qualitative methods [53, 58, 77]. In one study, caregivers had higher levels of anxiety than patients [40]. Caregivers reporting higher levels of unmet needs were more likely to report subclinical or clinical anxiety and/or depression [39]. In this study, the healthcare and psychological domains of the SCNS-P&C showed the strongest association with caregiver anxiety and depression. A number of individual items were also found to be associated with either anxiety or depression: carer anxiety was most strongly associated with caregivers needing help finding meaning in the patient’s illness, decision making in uncertainty and needing information about the patient’s prognosis. While depression was most strongly associated with needing to talk to other cancer carers, needing help with knowing how to discuss cancer socially and needing help with feelings about death.

A large population and registry-based cohort study in Denmark which included 5,774 partners of pancreatic cancer patients, found a higher incidence of first depression, anxiety and insomnia in this population compared to cancer-free spouses [63]. In contrast, the majority of caregivers in another study showed low levels of depression, with 32% scoring highly [72]. However, this was a much smaller study of 64 family caregivers of those with advanced pancreatic cancer who were newly enrolled in hospice care [72]. Evidence of anxiety and depression was also alluded to in qualitative literature for example, some caregivers reported use of medication to manage their anxiety and depression [58]. One caregiver commented: “I’m nervous… I have anxiety anyway, my anxiety just has been through the roof, pretty much out of control. I have panic attacks. My anxiety is just doubled or tripled I think which makes everything more difficult… Sometimes I’m just kind of collapsing and just emotionally I’m just frozen… I sleep instead of getting stuff done sometimes. If I have time I’ll nap and stuff instead of accomplishing something” [77].

Quality of life

Caregiver’s QoL was referenced within five included studies; three quantitative [40, 64, 68], one mixed methods [77] and one qualitative [45]. Caregiver’s QoL has been reported in relation to other domains of ‘The Cancer Family Caregiving Experience’ model including the negative impact of the patient’s GI symptoms on caregiver’s QoL [45], and the impact of positive coping on caregiver’s QoL [68]. A reciprocal relationship between patient and caregiver QoL was also alluded to in one study which found that higher patient health-related QoL was associated with higher caregiver health-related QoL at six-months post curative surgery [64]. Another study found that 58% of caregivers scored lower than the population average on a measure of QoL [40]. Finally, when individual aspects of QoL were analysed, emotional burden and low levels of positive adaptation were rated as having the greatest impact on caregiver QoL while financial concerns and disruptions to daily life were least impactful [77].

Other impacts on health and wellbeing

Other impacts on caregiver’s health and wellbeing including their overall health, levels of distress, loss of appetite, physical symptoms, and substance abuse were referenced within seven included studies; three quantitative [38, 66, 71], two qualitative [48, 53], and two mixed methods [73, 74]. Quantitative data suggested that spouses of patients with cancer experienced increased risk of several psychiatric disorders (depression, substance abuse, stress-related disorder) compared to spouses of people without cancer, within this pancreatic cancer spouses ranked the 3rd highest risk out of 21 cancer diagnoses (oesophageal and lung cancer were the only cancers to rank higher) [66]. In addition, the significant others of patients with pancreatic cancer reported higher distress scores than patients [71].

One study found that caregivers’ experience of powerlessness and hopelessness during palliative home care resulted in both physical and psychological symptoms such as muscle tension, loss of appetite, headache, anxiety, and depression [74]. Two case studies reported the experience of bereaved spouses [48, 53]. One reported on a woman who experienced dissociative amnesia largely due to trauma experienced from the unexpected death of her husband to pancreatic cancer [48]. The other found the bereaved wife of a pancreatic cancer patient, who fulfilled DSM-5 criteria for uncomplicated bereavement, to exhibit symptoms of anxiety, depression, and a decreased appetite which led to thiamine deficiency [53].

#### Disease trajectory

Many studies are able to be mapped to the disease trajectory, as proposed in the ‘Cancer Family Caregiving Experience’ model [23], however some fall outside of this model. For example, three included studies could be considered to focus on ‘Cancer Diagnosis/ Initial Treatment’; one focused on experience during treatment with curative intent [50], another during neoadjuvant therapy [77], and finally shared decision-making regarding surgery [43]. Another six studies focus on ‘Remission’ and ‘Surveillance’: one included those who had had surgery [47], while five studies focused on experiences following treatment with curative intent [36–38, 44, 64].

**Fig. 3:**
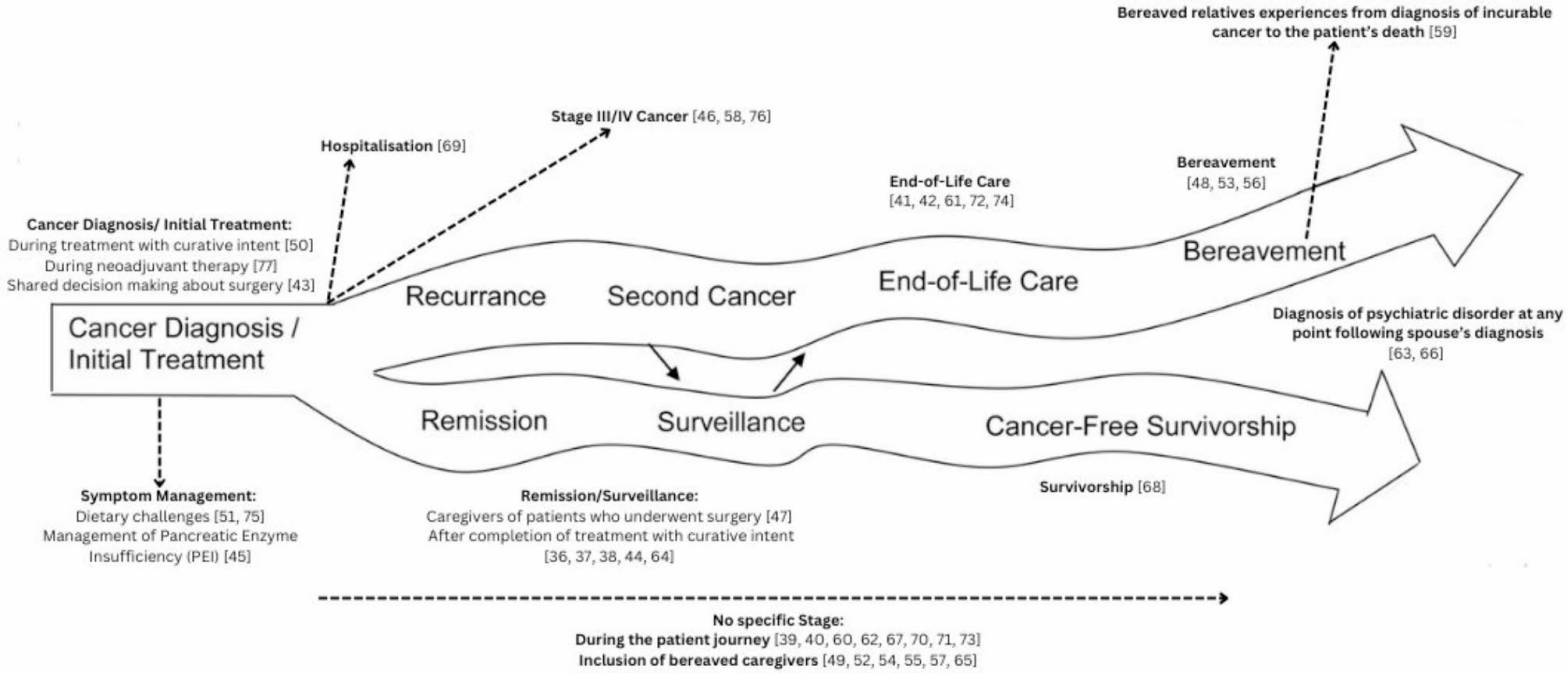
Cancer trajectory proposed within the ‘Cancer family caregiving experience’ conceptual model [23]

Other studies focused on factors which may branch off the proposed model. For example, those which focused on specific symptom management throughout disease progression rather than at one time point. Two such studies focused on dietary challenges [51, 75], while another focused on the management of pancreatic enzyme insufficiency [45]. Three studies included caregivers of patients with advanced (stage III/IV) cancer only [46, 58, 76]. Figure 3. presents the included studies as mapped to the proposed trajectory [23].

The influence of the stage in disease trajectory on the psychosocial impact experienced by caregivers was suggested across some included studies. Due to the unexpected diagnosis of advanced cancer, caregivers felt they had less time to process their emotions and were pre-emptively distressed with how they would cope with bereavement [60]. For example, one caregiver commented: “The fear of the unknown, the fear of not being sure of how it’s going to happen and how I’m going to react… I’m afraid of losing him…I’m worried about how I’m going to feel…What the hell is gonna happen to me? I have to stay here” [60]. Caregivers had difficulty accepting the often-incurable diagnosis and despite feeling they had been in a constant state of mourning, the time of the final loss remained difficult [55]. Some caregivers discussed that they had not had an opportunity to speak about their partners’ approaching death with them and although most accepted this as it was their partners wishes, some reported regret [59].

In terms of diagnosis news delivery, caregivers accepted that there was a rush due to the workload of healthcare professionals but emphasised that briefing should take place in a quiet, private area, with enough time to process information and ask questions [55]. Another study highlighted negative experiences of diagnosis: “When the actual first diagnosis was given to us, we had an unfortunate experience as it was rather brutal”, “I didn’t feel that the manner of delivery [of diagnosis] was particularly warm. It’s almost like some of the specialists are so experienced with what they’re doing that they lose sight of the fact that for the person who’s been diagnosed, it’s the first time they’ve heard these words” [57]. Caregivers suggested that clinicians should have a protocol to follow: “That conversation regarding pancreatic cancer is never going to be a nice one and we have to avoid shooting the messenger there. Maybe there could be some protocol that specialists follow a little more thoroughly. No-one is ever going to want to hear it so it’s never going to be well received” [57].

A number of time periods were identified as particularly stressful phases of caregiving. These varied for different patient populations. Caregivers of those who underwent pancreatectomy surgery identified the initial diagnosis, immediately following pancreatectomy surgery, following discharge from hospital, and during chemotherapy treatment as the most stressful time periods [38]. Another study with caregivers of post-operative patients found that caregivers described discharge from hospital as a heavy and stressful stage [47]. In comparison, caregivers of those with advanced pancreatic cancer described the period of time while the disease progressed, and the reality of the diagnosis took hold as extremely distressing due to a lack of control and often a sense of failure [76]. Additionally, as family members wished to spend more time with the patient during this time, some primary caregivers felt a lack of privacy and invasion of their personal space, especially when the patient’s needs increased, and their final days approached [76].

Disease trajectory was also found to influence caregiver’s perceptions of their unmet needs as caregivers of patients within four months of diagnosis had a higher prevalence of unmet needs than those who were 5–9 months post-diagnosis [39]. In one study, caregivers seemed to associate the word ‘cancer’ with death and found it difficult to make plans for the future, despite the fact the patients they cared for had undergone curative surgery [50]. The time of diagnosis was described as a ‘crisis stage’ with caregivers reporting feeling shocked, overwhelmed, and lost in the healthcare system [54, 76]. This shock was heighted if the patient had been perceived to live a healthy lifestyle prior to diagnosis [76]. Caregivers experienced a significant transition period when it became clear to them that the patient’s symptoms were caused by the disease rather than the treatment as this was accompanied by a realisation that the disease could not be cured. One caregiver described this as “cancer showing on the outside” [76].

Differences were found in coping between relatives of patients who were eligible for chemotherapy compared to those who were not [59]. When the patient was not eligible for chemotherapy, relatives described this as providing clarity about the incurable nature of the disease and so accepted this more quickly than relatives who hoped chemotherapy could extend their loved one’s life [59]. Planning ahead was discussed as a coping strategy by caregivers of patients with a terminal diagnosis. For example, 33% of caregivers in one study reported end-of-life planning as a coping strategy [77]. Some family caregivers of patients undergoing home-based palliative care inquired about funeral procedures [61]. Differences were also found in levels of dyadic coping based on the patient’s stage of disease for those who had undergone curative surgery, as levels of dyadic coping were found to decrease from baseline to 9-month follow-up, perhaps due to the patient requiring less intensive care as time moved on following surgery [64].

Caregivers of patient’s experiencing end-of-life care discussed an appreciation for spending more time together as comments included: “The end of life. There’s nothing good about his eventually dying. There’s no upside to that. There’s certainly a lot of good to my having this time with him. That’s a great thing” (son of a pancreatic cancer patient) [41]. While spouses in another study discussed the importance of having the time to spend together as a couple or with their children and/or grandchildren in comparison to those who have a sudden death: “Sometimes it happens that someone dies suddenly… and then you haven’t had that exhausting time, but at the same time you haven’t had the time to say goodbye to each other. And the knowledge [that the disease is terminal], that is really tough and painful, it’s really hard… but… you are able to say goodbye to each other” [59].

Finally, differences were observed in health and wellbeing outcomes dependent on disease trajectory. Bereaved caregivers were found to be at an increased risk of first depression while no differences were found in depression incidence based on tumour location or an advanced vs. non-advanced pancreatic cancer diagnosis [63]. Similarly, another study found no correlation between caregiver distress, perceived burden, anxiety, or depression with treatment intent (curative vs. palliative) or if the patient was undergoing active treatment or not [70]. On the other hand, time since diagnosis was found to impact risk of depression. The increased risk of first depression in pancreatic cancer partners compared to cancer-free spouses seen in the first year after diagnosis decreased with time so that 5 years following diagnosis, there was no longer a statistically significant difference in risk between partners of pancreatic cancer patients and cancer-free spouses [63]. One study of caregivers of patients who had undergone curative surgery found that most had depression and anxiety scores within the normal range throughout follow-up care [64]. 25% of caregivers in this study reported mild anxiety at first follow-up appointment which was maintained at nine-month follow-up [64]. Similar levels were found in another study of caregivers of post-operative patients in which 17.5% of caregivers scored positively for depression while 23.8% scored positively for anxiety [38].

#### Contextual factors

Some influence of contextual factors was observed within the included studies. For example, in one study, it was found that family members who lived in closer proximity to the patient were impacted by expectations from other family members to assume the primary caregiving role [76]. The primary caregiver often became the conduit of medical information to their wider family and friends and reported that this created additional tension and conflict between them. Those who lived further away reported a feeling of relief that they did not have direct responsibility for caregiving while on the other hand this led to a sense of lacking control over the situation.

Struggles with gender roles were also highlighted in one study which found male caregivers struggled to take on the role of meal preparation, especially when this was something they had not previously engaged with [51]. In another study, female caregivers, predominantly, described feelings of guilt and selfishness for taking time away from the patient [60]. While male caregivers were found to be more likely than female caregivers to report unmet needs [39]. Although no gender differences were found in the overall incidence of first depression in a study of 5,774 partners of pancreatic cancer patients, male partner’s risk of first depression remained higher for longer than women [63]. This study also found that shorter education was associated with increased risk of first depression.

In terms of age, younger caregivers (those under 60) were more likely to have higher levels of anxiety, depression perceived burden and poorer QoL than older caregivers [40]. Although another study found partners of older pancreatic cancer patients (aged 70 or older) were at a higher risk of depression compared to cancer-free spouses, the authors noted that this is likely due to the lower incidence of depression in this age group of the general population [63].

Differences in preferred place of death were discussed based on contextual factors, although positives were described of all settings (home, hospital, hospice) [42]. For example, when a loved one died in hospital due to their rapid deterioration, their family members felt that this was for the best due to the nursing care available. The bereaved daughter of pancreatic cancer patient discussed how her mother had previously had a good experience in hospice and so wished to die there, as she commented “they were so respectful in terms of our sort of traditions. It was just, it was like the perfect place for her to have been”. While a husband whose wife had died in hospice explained: “We had quite a nice time there actually… I was no longer worrying about being on top of any medicine that she required or painkillers… I had more time… [the patient] was comfortable. So, to say we had a nice time sounds really daft. But it was a… a good time” [42].

Finally, many similarities were observed between the findings of studies from the range of countries included within this review. For example, unmet needs, particularly the need for more information, were evident across European [46, 47, 55, 73], Australian [39, 45, 49] and American [56, 76, 77] studies. Impacts to employment and finances were referenced in several studies conducted in the USA [36, 38, 65, 76, 77] and one study conducted in China [69]. The impact of caregiving on anxiety and depression was also evident across European [63, 64, 66], Australian [39, 40], American [38, 65, 70, 72] and Asian [53, 67, 69] studies.

## Discussion

This scoping review aimed to examine the psychosocial impact of pancreatic cancer on caregivers across the disease trajectory and identify gaps in the literature. The main findings of the 42 studies included are discussed below in relation to the three main elements of the ‘Cancer Family Caregiving Experience’ conceptual model [23]: the stress process, cancer trajectory, and contextual factors.

The stress process model proposed within the ‘Cancer Family Caregiving Experience’ conceptual model consists of five constructs: primary stressors, secondary stressors, appraisal, cognitive-behavioural responses, and health and wellbeing [23]. This model enabled the synthesis of a broad range of literature within this review to provide greater insight and understanding of the psychosocial impact of pancreatic cancer on caregivers. Although the constructs were not always explicitly measured, evidence for each was found within the included studies. Appraisal was most commonly evident as included studies often focused on perceptions of caregiver burden, and unmet needs. Findings related to health and wellbeing outcomes, and secondary stressors were least common.

Primary stressors evidenced within the included studies included caregiving activities, and the impact of the person with pancreatic cancer’s symptoms. Difficulties with the range and volume of tasks were evident and exacerbated by feelings of helplessness and a lack of support [44, 45, 47, 55, 56, 60]. Despite their increasingly significant role in cancer patient’s care, and the documented toll this takes on their own health, cancer caregivers continue to report a lack of support [79–81]. Within the context of pancreatic cancer, this is likely heightened by the sudden diagnosis and short timeframe in which caregivers may be expected to learn new skills necessary to support the person with pancreatic cancer while they also come to terms with the diagnosis of their loved one [22].

Gastrointestinal symptoms such as diet restrictions and pancreatic exocrine insufficiency appeared to be a particular area of concern for caregivers [38, 49, 51, 58, 60, 75], and therefore may be important to consider in support tailored to this population. Caregivers’ own psychological wellbeing was also influenced by the person with pancreatic cancer’s [40, 70]. Such a reciprocal relationship between patient and caregiver emotional distress has been highlighted within other cancer patient-caregiver populations [82–85]. This may suggest support interventions for either the patient or caregiver could benefit both parties as well as the utility of dyadic or family-based interventions. Additionally, the high psychological symptom burden experienced by those with pancreatic cancer may heighten this reciprocal relationship [8–10].

Common secondary stressors were changing roles and relationships within families. Spending more time together during the illness period potentially brought families closer together or sometimes exacerbated pre-existing negative family dynamics [54, 76]. In the broader adult cancer caregiving research, there has been limited attention placed on wider family impacts, with a predominated focus on the primary caregiver. However, similar impacts have been evidenced within dementia care [86, 87]. Other stressors included a loss of working hours and productivity which led to financial difficulties [36, 38, 65, 69, 73, 76, 77]. A previous review of the financial costs of cancer caregiving found the costs of caregiving (e.g. time spent providing care, medical appointments, household activities) averaged between $2,800 - $4,800 a month and were highest during the palliative phase [88]. Impacts to employment may vary for pancreatic cancer caregivers, depending on disease trajectory. For example, it has been suggested that caregivers of people who underwent pancreatectomy experienced a loss in work productivity more similar to caregivers of those who experienced traumatic orthopaedic injury than those with other advanced cancers or chronic diseases [36].

Appraisal of stressors was often evidenced as unmet needs, particularly in relation to healthcare services and information needs, for example due to a lack of information about treatment options, prognosis, and symptom management [39, 46, 47, 49, 56, 73, 77]. This mirrors the findings of a recent systematic review of unmet needs among pancreatic cancer caregivers [22] and within the wider advanced cancer caregiver population [21]. Some perceived rewards and benefits were reported such as an appreciation for spending more time with the patient and finding strength in their ability to provide care [41, 52, 59, 76]. Such rewards have been reported previously in palliative care literature [89, 90] and therefore may reflect the fact that the majority of pancreatic cancer diagnoses are received at a late stage.

Cognitive and behavioural responses included coping strategies such as hiding feelings to appear strong and developing caregiving skill [44, 49, 54, 75–77]. This highlights a potentially missed opportunity to support caregivers to develop their skill as they sought to do so themselves. Accessing support services was rarely mentioned and when it was, caregivers reported finding support services themselves, rather than being signposted to these [49]. Coupled with hiding emotions, this may suggest this population lack support or are unaware of the support services they may avail of. Caregivers often shared hopes for how services may be improved including provision of an opportunity for caregivers to discuss their concerns with a healthcare provider [43, 44, 55, 57, 76, 77].

The need for increased communication between healthcare providers and caregivers is highlighted by pancreatic cancer caregivers suggestions of a ‘caregiver consultation’ [44], along with the reported levels of unmet needs within healthcare services and information needs as well as many caregivers feeling unsupported throughout the included literature. Feasibility studies of interventions which aim to address this have shown some promise, for example, a telemedicine intervention in a palliative care setting which allowed patients and caregivers to easily contact their physician via video call improved both patient and caregiver QoL [91]. Cancer caregivers also favourably rated an app which aimed to improve caregiver’s communication skills, facilitate information sharing with other family members, provide resources for self-care, and increase knowledge about disease management [92]. Future evaluation and implementation of such interventions may benefit pancreatic cancer caregivers.

Health and wellbeing outcomes such as anxiety, depression, and QoL were evident across the literature. In line with previous reviews regarding cancer caregivers [81, 93, 94] and specifically pancreatic cancer caregivers [5, 22], negative impacts on QoL were evident with pancreatic cancer caregivers experiencing lower QoL than the population average [40]. Pancreatic cancer caregivers were also at increased risk of several psychiatric disorders compared to caregivers of people with other cancers (outranked only by oesophageal and lung cancer) [66]. This likely reflects the poor survival rates associated with these cancers [95] and emphasises the need for timely psychosocial support for this population. For example, the emotional support provided by a counselling intervention in an Australian feasibility study which has been rated as beneficial by both pancreatic cancer patients and caregivers [96]. The risk of psychological morbidity within pancreatic cancer caregivers may be greater due to diagnoses most often occurring at an advanced stage at which point patients are not eligible for potentially curative treatment. This is emphasised by the fact that included studies with caregivers of patients who had been eligible for surgery showed lower levels of anxiety and depression [64]. This highlights the need for timely psychosocial support, tailored to meet the needs of these different populations of caregivers.

The ’Cancer Family Caregiving Experience’ conceptual model proposes that the stress process occurs at any point across the disease trajectory but is likely experienced differently during different phases (e.g. at the time of diagnosis or end-of-life care) [23]. The lack of longitudinal studies limits conclusions on how the psychosocial impact of pancreatic cancer on caregivers may change across the illness trajectory, however the influence of disease trajectory on the pancreatic cancer caregiver experience was referenced within some studies. For example, caregivers of people with pancreatic cancer within four months of diagnosis had a higher prevalence of unmet needs than those who were 5–9 months post-diagnosis [39]. This is in contrast to a previous longitudinal study with people with pancreatic cancer whose unmet needs persisted over a similar time period [97] and highlights the differing experiences between caregivers and people with pancreatic cancer. Caregivers’ unmet needs may be particularly high in the period immediately following diagnosis as they cope with the diagnosis of their loved one and are thrust into their new role. This time period is often characterised by new caregiving demands and substantial lifestyle changes which caregivers may adjust to over time [98].

As pancreatic cancer is most often diagnosed at an advanced stage and survival rates are low [1–3], the disease trajectory may be accelerated. Therefore, the pancreatic cancer journey may not map directly to a broader cancer trajectory. In addition, due to the poor prognosis, with most people being ineligible for potentially curative treatment [2], a large proportion of cases may skip aspects of the trajectory such as ‘initial treatment’, and ‘recurrence’ while few will progress through the pathway to ‘cancer-free survivorship’. Nine of the included studies focused on aspects of treatment with curative intent and as only a small percentage (around 20%) of people diagnosed with pancreatic cancer are eligible for such treatment [99] it is likely that experiences between these two groups differ. Additionally, the majority of included studies did not differentiate between stages of disease. Future research may benefit from differentiating the experiences of caregivers across different illness trajectories, in which the psychosocial impact may vary, to explore their potentially differing needs.

Some evidence of the influence of contextual factors was observed in the included studies, however data in this area was limited mainly to gender and age. Consistent with exiting literature [80, 94], younger caregivers reported higher levels of anxiety, depression, and perceived burden, and lower QoL [40, 70]. Male caregivers may be more likely to report unmet needs [39] and their risk of first depression remained higher for longer than women [63]. A previous systematic review including cancer caregivers contrastingly found female caregivers to report higher levels of distress and unmet needs than male caregivers [100, 101]. Another study included in this review may help to explain this difference as it appeared gender norms regarding meal preparation caused some distress as male caregivers struggled to take on this role especially when it was something they had not previously engaged with [51]. This may reflect the dietary challenges associated with pancreatic cancer which appeared to be a particular area of concern for pancreatic cancer caregivers throughout the included literature.

### Strengths and limitations

Strengths of this scoping review include the rigorous methodological approach which followed internationally recognised methodology (JBI) for the conduct of scoping reviews. Additionally, the use of the ’Cancer Family Caregiving Experience’ conceptual model to guide the analysis of results of the included studies in this review enabled the results of a large number of studies of differing methodology to be organised and interpreted in relation to the wide body of international cancer caregiving literature [23]. The model considers many factors which influence the cancer caregiving experience [23], and although each included study did not always explicitly map to the model, it’s comprehensive structure enabled the integration of a wide range of factors to facilitate a holistic understanding of the pancreatic cancer caregiving experience. However, some aspects of caregiving may not be fully addressed within this model. For example, although the impact of the patient’s psychological wellbeing on that of caregivers was considered, dyadic models may better account for such reciprocal relationships between caregivers and patients. Additionally, the model may not capture the varying experiences of different cancer trajectories. Specifically, within the case of pancreatic cancer, where the proposed disease trajectory may be accelerated.

The included quantitative studies most often examined ‘health and wellbeing’ outcomes such as anxiety and depression, offering measurable insights into the psychosocial impact of pancreatic cancer on caregivers. In contrast, the included qualitative studies explored ‘stressors’ such as caregiving activities and ‘appraisal’ such as future outlook and discussion of unmet needs. Qualitative data helped to provide meaning to the quantitative data presented and when integrated within the domains of the ‘Cancer Family Caregiving Experience’ model, both methodologies helped to provide a comprehensive understanding of the psychosocial impact of pancreatic cancer on caregivers. When developing support for pancreatic cancer caregivers, it is essential that findings from both quantitative and qualitative studies are considered to ensure that interventions meet the multi-faceted needs of pancreatic cancer caregivers.

This scoping review did not include a formal quality appraisal of the included studies in line with methodological recommendations for systematically conducting scoping reviews [102, 103]. Although the aims of this review were to synthesise existing evidence rather than assess the quality of existing studies, the absence of quality appraisal limits the ability to draw conclusions regarding the methodological rigor of the included studies. Other limitations of this review, include restrictions placed on the language of included studies as it is likely important evidence, not in the English language, may have been missed. However, the number of studies included may be regarded as a strength, as this incorporated studies from different countries, caregiver populations, and methodologies. On the other hand, the heterogeneity in methods of the included studies may have limited the comparison of study findings. However, the similarities of the outcomes of many of the included studies across countries and cultures, despite the differing methodologies used, highlights the potential for collective interpretation of the included studies.

Although, similar outcomes were observed between many included studies, conclusions may be limited by the differing methodologies used between studies as well as due to differing contextual factors, particularly culture. Previous research has highlighted that cancer caregivers’ needs are influenced by their cultural values (e.g. between Eastern and Western cultures) [21, 104]. However, cross-cultural explorations are not possible within the present review as unmet needs were not explicitly examined in any studies conducted in typically Eastern cultures. Additionally, cross-cultural comparisons are limited regarding the financial impact of caregiving. Although evident within several American studies [36, 38, 65, 76, 77], data regarding employment and finances was limited in relation to other countries and only observed within one study conducted in China [69]. This may reflect the fact that caregivers in the USA potentially face increased financial burden [105]. Future research may benefit from considering contextual issues present within the study population and conducting cross-cultural studies to directly compare the experiences of pancreatic cancer caregivers in different countries.

In terms of the literature included in this review, the majority of studies were cross-sectional and so inferences regarding changes in the caregiving experience across the disease trajectory are limited. In addition, the majority of participants across the included studies were female and the partner of the person with pancreatic cancer, while 16 of the 42 studies were conducted in the USA. This lack of diversity may limit the generalisability of the results. Another limitation of this review was the lack of consistent caregiver definitions across the included studies. For example, not all studies provided inclusion criteria for caregivers and those that did varied and included self- and patient-identified caregivers, spouses identified from medical records, next of kin or relatives, or specific criteria such as: an adult family caregiver who had cared for the person with cancer for more than three months. Further, the majority of studies included pancreatic cancer caregivers among caregivers of other cancers and/ or patients which lead to a smaller amount of data being relevant to this review as pancreatic cancer caregivers were not the main focus of the existing literature.

The included literature often focused on one aspect of the disease or stage of the disease trajectory, therefore there exists a need for future longitudinal research to explore the pancreatic cancer caregiver experience over time from pre-diagnosis to survivorship or bereavement. This may be especially true for pancreatic cancer in which the disease trajectory can differ substantially from other cancers. In addition, future research should focus specifically on pancreatic cancer caregivers to allow their unique experiences to be explored in-depth. This is emphasised by the challenges highlighted in this review which may be unique within the pancreatic cancer caregiving journey such as the impact of gastrointestinal symptoms. Additionally, as pancreatic cancer caregivers are at an increased risk of psychiatric disorders [63, 64, 66, 72], screening processes which enable early intervention may be beneficial to this population. Coupled with the feelings of a lack of support reported further highlight the need to prioritise signposting caregivers to existing support services such as counselling, and peer support. The findings emphasise the need for tailored interventions which address the multi-faceted needs of pancreatic cancer caregivers.

### Conclusions

Pancreatic cancer caregivers commonly experience stress related to caregiving activities, changes in their daily life and family relationships, financial difficulties, high levels of unmet need and distress, and are at risk of several psychiatric disorders. Overall, the psychosocial impact of pancreatic cancer on caregivers shares similarities with that of other cancer caregivers although there are some notable differences. For example, difficulties with the caregiving role, and substantial lifestyle changes in a short time period which are likely heighted by the nature of pancreatic cancer. Feelings of a lack of support were reflected throughout the included literature and emphasise the need for future research into how pancreatic cancer caregivers may be best supported, and sign-posted to existing support, to minimise the substantial psychosocial impact they may experience.

## Electronic supplementary material

Below is the link to the electronic supplementary material.

Supplementary Material 1– MEDLINE Search Strategy

Supplementary Material 2– Included Study Characteristics

## Data Availability

No datasets were generated or analysed during the current study.

## Abbreviations

QoL: Quality of Life
ScR: Scoping Review
JBI: Joanna Briggs Institute
PRISMA-ScR: Preferred Reporting Items for Systematic Reviews and Meta-analyses extension for Scoping Reviews
