# Supplementary material for: The psychosocial impact of pancreatic cancer on caregivers: a scoping review
Source: BMC Cancer. 2025 Mar 20;25:511. doi: 10.1186/s12885-025-13891-w (PMC11924831; doi:10.1186/s12885-025-13891-w)
Supplement: Supplementary file 1 — Supplementary Material 1– MEDLINE Search Strategy [file 12885_2025_13891_MOESM1_ESM.docx]

Supplementary Material 1.

Example Search Terms Applied in MEDLINE

| **#** | **Query** |
| --- | --- |
| **MEDLINE** | |
| 1 | exp Pancreatic Neoplasms/ |
| 2 | (pancreatic-cancer OR pancreatic-neoplasms OR pancreatic-adenocarcinoma OR cancer-of-the-pancreas).mp |
| 3 | 1 OR 2 |
| 4 | exp Caregivers/ |
| 5 | (Caregiv* OR carer* OR famil* OR spouse* OR partner* OR next-of-kin OR relative OR son* OR daughter*).mp |
| 6 | 4 OR 5 |
| 7 | (quality-of-life OR psychological-need or physical-need or informational-need OR psychological-distress OR stress* OR burnout* OR experience* OR depression OR anxiety* OR emotion* OR wellbeing OR burden* OR concern* OR worry OR coping* OR perception* OR relationship*).mp |
| 8 | 3 AND 6 AND 7 |
| 9 | limit 8 to English language |
