# Supplementary material for: The psychosocial impact of pancreatic cancer on caregivers: a scoping review
Source: BMC Cancer. 2025 Mar 20;25:511. doi: 10.1186/s12885-025-13891-w (PMC11924831; doi:10.1186/s12885-025-13891-w)
Supplement: Supplementary file 2 — Supplementary Material 2– Included Study Characteristics [file 12885_2025_13891_MOESM2_ESM.docx]

Supplementary Material 2.

Characteristics of Included Studies

| Type | Author (year), country | Phenomena of interest | Study Design | Caregivers’ characteristics | Methods | Outcomes of interest |
| --- | --- | --- | --- | --- | --- | --- |
| Mixed methods (*n* = 5) | Grivel et al. (2023), France [73] | The experience of caregivers or patients with digestive cancer | Cross-sectional prospective with qualitative dimension | 7 pancreatic cancer caregivers (32 total)  Relationship to pancreatic cancer patient: 1 child, 4 spouse, 1 sibling, 1 in-law  Aged 20-79 | Questionnaires: Caregiver Reaction Assessment (CRA), Supportive Care Needs Survey for Patients and Caregivers (SCNS-PC)  Semi-structured interviews | Unmet supportive care needs, high caregiver burden  Qualitative themes:  ‘Illness is an upheaval in the caregiver’s life’, ‘He feels alone and helpless dealing with the disease and suffers its consequences’, ‘Despite this, he naturally assumes his role while managing to remain positive’ |
|  | Milberg et al. (2004), Sweden [74] | The experience of powerlessness and helplessness in next of kin of cancer patients in advanced palliative home care | Cross-sectional mixed methods | 20 pancreatic cancer next of kin (233 total)  Patients were either currently experiencing palliative care or had died 3-9 months previously (after experiencing palliative home care) | Postal questionnaires with Likert-type and open-ended questions focused on powerlessness and helplessness | Powerlessness and helplessness common among this population |
|  | Milliron et al. (2023), USA [75] | Caregiver preparedness, self-efficacy, barriers and facilitators to healthful eating among gastrointestinal patients and their caregivers | Concurrent explanatory mixed-methods cross-sectional study | 27 patient-caregiver dyads (56% pancreatic cancer)  59% female. 93% spousal dyads, 7% parent-child | Structured surveys (self-efficacy scale for managing cancer symptoms, preparedness for caregiving scale, healthy eating index [HEI-2015]) and dietary assessments.  In-depth interviews | Caregivers had low self-efficacy in managing treatment side effects and felt unprepared for the stress of caregiving |
|  | Ruff et al. (2023), USA [77] | Caregiver wellbeing during neoadjuvant therapy (NT) for patients with localised pancreatic cancer | Cross-sectional mixed methods | 28 pancreatic cancer caregivers  Mean age 60.1, 75% female, 71.4% spouse, 14.3% child, 7.1% sibling | Caregiver quality of life index-cancer (CQOLC) survey  Semi-structured interviews | Emotional burden and positive adaptation had greatest negative impact on QoL index while financial concerns and disruption to daily life were less impactful  Qualitative themes:  Caregiver perspective of treatment and patient experience  Caregiver emotional symptoms  Impact on caregiver daily life  Caregiving coping  Caregiver goals, needs, and recommendations |
|  | Sherman et al. (2014), USA [76] | The experience of family caregivers of patients with advanced pancreatic cancer | Mixed methods pilot study | 8 pancreatic cancer caregivers  Aged 37-74, 87.5% female, 62.5% spouse, 25% child of patient, 12.5% mother of patient | Measured caregiver strain, mastery, symptoms, post-traumatic growth, & QoL  Follow-up interviews | Themes highlighted included: crisis of diagnosis, violation of assumptions about life and health, and unmet caregiver needs |
| Qualitative (*n* = 22) | Block (2001), USA [41] | “Best possible” scenario for end of life | Case report | Son of pancreatic cancer patient  Lives 2,000 miles from patient, pancreatic cancer diagnosis 2 years ago | Present an “extraordinary case” of a “best possible” scenario | Themes:  Perspectives (benefit of having time together),  Maintaining a sense of continuity for one’s self (appreciating the day-to-day things),  Confronting and preparing for death (“at some age it’s going to happen to everybody”) |
|  | Chapple et al. (2011), UK [42] | The preferred place of death for pancreatic cancer patients reaching the end of life | Qualitative interview study | 8 bereaved relatives of pancreatic cancer patients  Age ranged from 35 to 74, 37.5% female | Semi-structured interviews | Themes:  Care available at home (e.g. palliative care nurse),  Experience of hospital care,  Perceptions and experiences of hospice care |
|  | Geessink et al. (2017), Netherlands [43] | Optimal treatment decision-making for surgeons and older patients with colorectal or pancreatic cancers | Qualitative focus group study | 7 pancreatic cancer relatives (14 total)  All patients over the age of 65 and diagnosed within last 5 years  71.4% spouse, 14.3% child, 14.3% neighbour  Age ranged from 68-83  57.1% female | Focus groups with older patients and their relatives | Themes:  Emotions and coping styles,  Patient’s mental capacities,  Trustworthiness (of physicians),  Third person (support from a third person – family member – during a consultation helpful) |
|  | Gerhardt et al. (2020), Denmark [44] | Experiences of caregivers of patients attending follow-up after completion of treatment with curative intent for cancers of the pancreas, duodenum and bile duct | Qualitative interview study | 7 pancreatic cancer caregivers (10 total)  60% female  70% spouse, 10% sibling, 20% adult children | Semi-structured, individual interviews | Themes identified: ‘From bystander to enlisted care’, ‘lonesome worrying’, ‘Keeping a stiff upper lip’ |
|  | Gooden & White (2013), Australia [45] | Supportive care needs during management of pancreatic exocrine insufficiency | Qualitative inquiry framework | 23 pancreatic cancer caregivers  87.0% female, age ranged from 20 to 79  Majority current caregivers (1 bereaved who had lost both parents to pancreatic canver) | Semi-structured interviews by telephone or face-to-face | Management of pancreatic exocrine insufficiency caused distress and negatively impacted caregiver QoL  Increased information identified as a supportive care need |
|  | Griffioen et al. (2021), Netherlands [46] | How experiences of patients, their significant others, and medical professionals over the entire care path accumulate to support their ability to participate in shared decision-making | Qualitative interview study | 13 significant others of pancreatic cancer patients | Interviews based on a service design perspective | Themes:  Decision making as an ongoing and unpredictable process,  Division of roles, tasks, and collaboration,  “Work” for the patient and/or significant other in obtaining and understanding information,  In “their disease journey” patients are confronted with unexpected energy drains and energy boosts, that influence their empowerment to participate in shared decision-making |
|  | Ibrahim et al. (2020), Sweden [47] | The experiences of participation among the next of kin of patients who had surgery for upper abdominal tumours | Qualitative interview study | 6 pancreatic cancer next of kin (11 total)  66.7% female, age ranged from 27-81, 66.7% child of patient, 33.3% spouse | Interviews conducted 1-3 weeks post-op | Two main themes: ‘from the shadows to an important role’ and ‘an inviting and inhibiting environment for participation’ |
|  | Ishida et al. (2015), Japan [48] | The development of dissociative amnesia in the recently bereaved | Case report | A 38-year-old woman attending a psychiatric consultation whose 44-year-old husband had died of pancreatic cancer a day prior | Present the psychiatric symptoms of a bereaved spouse. | Caregiver experienced dissociative amnesia on the day her husband died from pancreatic cancer, trauma of death considered main cause |
|  | Khan et al. (2022), Australia [49] | The supportive care experiences of patients and caregivers living with pancreatic and oesophageal cancers | Qualitative interview study | 7 pancreatic cancer caregivers (11 total)  90.9% female | Semi-structured interviews  Majority interviewed with the patient they cared for, others individual interviews (due to bereavement or patient being too ill to participate) | Three themes identified:  Inadequate support for symptoms and issues across the cancer journey,  Caregiver’s desire for greater support,  A multidisciplinary care team is the hallmark of a positive supportive care experience |
|  | Khatri et al. (2012), Australia [50] | Lived experience of patients with gastrointestinal cancer and their caregivers during treatment with curative intent | Phenomenological | 2 pancreatic cancer caregivers (6 total)  Both wives of pancreatic cancer patients, aged 51 and 52  Both patients had had Whipple’s procedure | Semi-structured individual interviews | Perception and experience of time was altered, themes included:  ‘Recall of intricate details’, ‘Waiting’, ‘Changing pace of time’, and ‘Being towards death’ |
|  | Locher et al. (2010), USA [51] | The social organisation of caring as gendered work in relation to meal preparation surrounding older adult cancer patients and their caregivers | Qualitative interview study | 30 caregivers of patients diagnosed with pancreatic, colon, breast, lymphoma, skin, and head and neck cancers (not reported how many are pancreatic cancer caregivers but their quotes have been labelled and extracted) | In-depth, semi-structured, face-to-face interviews | Males reported feeling helpless with meal preparation, some struggled with getting a pancreatic cancer patient to eat, caregivers struggled with female patients who were distressed by not being able to prepare their own food |
|  | Nolan et al. (2006), USA [52] | Spiritual issues addressed by users of a pancreatic cancer informational website | Qualitative descriptive | 19% of 600 postings included content about spirituality  68% of postings indicated that the poster was female  Majority from family members, 31% daughter, 9% wife (majority unclear) | Qualitative descriptive study of 600 internet postings downloaded from a patient and family chat room | Themes identified:  Spiritual convergence,  Reframing suffering,  Hope,  Acceptance of the power of god and eternal life |
|  | Onishi et al. (2019), Japan [53] | Thiamine deficiency in bereaved partners after spousal loss from cancer | Case report | A 57-year-old woman whose husband had died one year following a pancreatic cancer diagnosis | Present case reports of thiamine deficiency in bereaved spouses | Caregiver experienced anxiety, depression and decreased appetite following husband’s death  Loss of appetite lead to thiamine deficiency  Feelings of regret regarding choices made in relation to treatment options and place of care. |
|  | Petrin et al. (2009), USA [54] | The experience of family caregivers in communicating about and adjusting to a relatives diagnosis of pancreatic cancer, treatment, and subsequent survival or death | Qualitative interview study | 22 first-degree-relatives of pancreatic cancer patients  50% sibling, 31.8% child, 18.2% parent  45.5% female  50% bereaved | Telephone interviews | Themes:  Feeling surrounding news of diagnosis,  Coping in the aftermath of the diagnosis,  Addressing one’s own feelings,  Support system,  Family dynamics,  The future |
|  | Ploukou et al. (2023), Greece [55] | Informal caregivers’ experience of supporting family members with pancreatic cancer | Qualitative descriptive | 10 pancreatic cancer caregivers  70% female  30% spouse of patient, 60% child, 10% sister in-law  60% caregivers to patients undergoing chemotherapy, 40% bereaved caregivers | Individual semi-structured telephone interviews | Caregivers referred to their needs for information, psychological and financial support, education or support at caregiving activities, and communication with the patients |
|  | Rabow et al. (2004), USA [56] | Family caregivers experience at end of life | Case report | Wife and daughter of a pancreatic cancer patient who had died | Individual interviews with wife and daughter shortly after patient’s death from pancreatic cancer | Different information sought by different family members, caregiving helped family feel useful and closer to patient, felt they were not aware of what caregiving would entail at the beginning of their journey |
|  | Saunders et al. (2009), Australia [57] | The views of people affected by pancreatic cancer with regard to research priorities | Qualitative focus group study | 6 pancreatic cancer caregivers; 5 current and bereaved carers completed a focus group and an additional bereaved carer provided email responses  71.4% female  Females aged 30-49, males aged 60-69 | Teleconference focused discussion group | Themes identified for which participants would like to see increased research focus:  Early detection, clinician communication, public awareness, more and improved treatment options  The emotional experience (e.g. anger, frustration and worry) also discussed. |
|  | Tang et al. (2018), USA [58] | Symptom experiences in patients with advanced pancreatic cancer | Qualitative descriptive | 34 pancreatic cancer caregivers  61.8% spouse, 17.6% child, 8.8% sibling, 8.8% friend, 2.9% mother | Audi-recorded, naturally occurring encounters among advanced pancreatic cancer patients, caregivers, and healthcare professionals | Caregivers played significant role in symptom management and articulating symptoms to healthcare professionals.  Symptoms often distressing for caregivers. |
|  | Wijnhoven et al. (2015), Netherlands [59] | Bereaved relatives’ experiences from the time of diagnosis of incurable cancer until death with specific emphasis on their role in the end-of-life decision-making concerning chemotherapy | Qualitative interview study | 7 pancreatic cancer relatives (15 total)  50% female, average age of 59 years, 73% partner of patient.  Patients had died at least 6 months prior. | In-depth interviews | Themes:  Slow acceptance of the incurable nature of the disease,  When chemotherapy is starting to become useless,  Changing care roles throughout different stages of the disease |
|  | Wong et al. (2019), Germany [60] | The factors associated with psychological distress in pancreatic cancer from patient and caregiver perspectives | Photovoice | 7 pancreatic cancer caregivers  57.1% female  Age ranged from 50-69  85.7% partner of patient  85.7% caregiver to patient in active treatment, the remaining participants were undergoing surveillance | Group discussions using photovoice methods | Themes identified:  Diagnosis of an unexpected advanced cancer,  Management of weight loss and gastrointestinal symptoms,  Changes in role and identity,  Fear of the future |
|  | Zhang et al. (2023), China [61] | The needs and coping strategies of family caregivers in home-based palliative care | Field study | 2 pancreatic cancer caregivers (total 25)  Relationship to pancreatic cancer patient: 1 adult son, 1 wife | Semi-structured interviews, participant observation, documents and records collection | Funeral planning and use of web-chat support group discussed by pancreatic cancer caregivers |
|  | Zhang et al. (2024), China [62] | Disease coping experiences of pancreatic cancer patients and their spouses | Qualitative interview study | 10 spouses of pancreatic cancer patients  Median age: 63 (47-73)  Majority had a secondary school education or below  70% female | Semi-structured individual in-depth interviews | Five themes identified: denial and silence, fear and worry, struggle, coping strategies, cherishing the present |
| Quantitative (*n* = 14) | Dengsø et al. (2021), Denmark [63] | The psychological symptom burden in partners of pancreatic cancer patients | Population-based cohort study | 5,774 pancreatic cancer caregivers  41% female  Median age 66.7  72% partners of a patient with advanced pancreatic cancer | Medical records analysed for first onset of depression, anxiety, or insomnia following pancreatic cancer diagnosis of partner | Greatest risk of first onset of anxiety, depression and/or insomnia in first year following diagnosis  Bereaved partners at greater risk |
|  | Elberg Dengsø et al. (2023), Denmark [64] | Physical and psychological symptom burden in patients and caregivers during follow-up care after curative surgery for cancers in the pancreas, bile duct, or duodenum | Prospective observational cohort study | 54 pancreatic cancer caregivers (75 total)  65% female  Median age 71.1  89% spouse | Questionnaires measured health-related quality of life, anxiety, depression, dyadic coping, caregiver burden at baseline (follow-up appointment), 6-months and 9-months later | Patient and caregiver HRQoL more dependent on each other between baseline and 6-month follow-up, greater dyadic coping during this time period  Mild anxiety in caregivers  Low number of caregivers experienced high caregiver burden |
|  | Engebretson et al. (2015), USA [65] | Patient and caregiver perceptions about diagnosis and daily life with pancreatic cancer | Cross-sectional | 213 pancreatic cancer caregivers  89% female  111 current caregivers, 102 bereaved within previous 6 months | Questionnaires measured patients symptoms and diagnosis, emotional impact of pancreatic cancer, caregiver role, awareness and use of support services | Difficulty with diagnosis, anxiety and depression reported, low levels of positive outlook, caregivers concerned with patients’ quality of life, symptom management and extending life |
|  | Fong et al. (2022), USA [36] | Caregiving-related effects experienced by caregivers of patients with pancreatic and periampullary cancers who have had a pancreatectomy | Cross-sectional | 240 pancreatic cancer caregivers  Median age 60  70.8% female | Instrument developed based on National Study of Caregiving which measured social support and service use, work productivity, caregiving-related difficulties and participation effects | Loss in working hours and productivity due to caregiving which creates increased financial and emotional difficulties |
|  | Fong et al. (2023), USA^a^  [38] | Frequency and difficulty of specific assistance pancreatic cancer caregivers provide | Cross-sectional | 240 pancreatic cancer caregivers  Median age 60  70.8% female | Instrument developed based on National Study of Caregiving which measured caregiver wellbeing and support, nature and difficulty of assistance provided, most stressful time periods and caregivers’ needs | Depression and anxiety commonly experienced, immediately post-operative and early discharge identified as most stressful phases, desire for more information and better preparation |
|  | Fong et al. (2023), USA^b^  [37] | Caregiver-reported quality of communication with clinical team members in post-pancreatectomy period | Cross-sectional | 240 pancreatic cancer caregivers  Median age 60  70.8% female | Questionnaire: The Caregiver Perceptions About Communication with Clinical Team Members (CAPACITY) | Communication with care team rated better in regards to patient care but lower in supporting and integrating caregivers in decisions  Caregivers reported emotional, financial, and physical difficulties |
|  | Hu et al. (2023), Denmark and Sweden [66] | Risk of psychiatric disorders among spouses of patients with cancer vs spouses of individuals without cancer | Population-based cohort study | 546,321 spouses of patients with cancer (not clear how many of these were pancreatic cancer caregivers however findings are reported individually for each cancer, and so pancreatic cancer results have been extracted) | Medical records analysed for first clinical diagnosis of a psychiatric disorder compared to matched spouses to individuals without cancer | Increased risk of first-onset psychiatric disorder (depression, substance abuse, stress-related disorder) most prominent in spouses of individuals with cancer in the oesophagus, lung, and pancreas – pancreatic cancer showed third highest hazard ratio (out of 20 cancer types) |
|  | Huynh et al. (2023), Australia [39] | Unmet supportive care needs among carers of pancreatic cancer patients and if these are associated with anxiety and depression | Cross-sectional sub-study of a longitudinal repeated measures study | 84 pancreatic cancer caregivers  72.6% female  81% partner of patient, 12% child, 7% parent/friend/other | Questionnaires: Supportive Care Needs Survey for Partners and Caregivers (SCNS-P&C), HADS | Unmet supportive care needs associated with experience of anxiety and/or depression |
|  | Janda et al. (2017), Australia [40] | Anxiety, depression and quality of life in people with pancreatic cancer and their carers | Cross-sectional | 84 pancreatic cancer caregivers  72.6% female  81% partner of patient, 12% child, 7% parent/friend/other | Questionnaires: Hospital Anxiety and Depression Scale (HADS), Functional Assessment of Cancer Therapy for the general population (FACT-GP) | Elevated anxiety and depression, and reduced QoL seen in caregivers three months after diagnosis  Anxiety more common in carers than patients |
|  | Li et al. (2023), China [67] | The relationship of intimacy, dyadic coping, and psychological distress among pancreatic cancer and their spousal caregivers | Cross-sectional | 277 pancreatic cancer caregivers  58.8% female  Age ranged from 30 – over 60 | Questionnaires: Dyadic coping scale (DCI), Quality of Relationship Index (QRI), HADS | Pancreatic cancer patients and spousal caregivers cope interdependently, dyadic coping & intimacy associated with reduced anxiety and depression |
|  | Liu et al. (2023), China [68] | The interaction of positive coping style and quality of life between survivors of pancreatic cancer and their caregivers | Cross-sectional | 200 pancreatic cancer caregivers  Age ranged from 24-78  67.5% female  54% spouse, 35% child, 4.5% parent, 6.5% other | Actor-Partner interdependence model  Questionnaire: Simple Coping Style Questionnaire, 12-item Short Form Health Survey | Higher caregiver positive coping levels predicted both the individual QoL of the caregiver and the patient’s QoL |
|  | Sherman & McMillan (2015), USA [72] | Perceived social support, perceived health, and depressive symptoms of caregivers of hospice patients with advanced pancreatic cancer | Cross-sectional | 64 primary caregivers of patients with advanced pancreatic cancer newly admitted to home hospice care  Mean age 68.4, white 96.9%, female 68.7%, married 79.7%, 70.3% spouses or significant other of patient | Perceived social support (3-item measure by Krause & Borawski)  Self-rated Health Perceptions and Physical Functional Health (two subscales from the medical outcomes study short-form health survey)  Centre for Epidemiologic Study-Depression Short Form scale | Most satisfied with emotional support  Reported perceived health as fair to good  Low scores of depression, 32% scored high |
|  | Sun et al. (2023), China [69] | Levels of care burden and its influencing factors of caregivers of pancreatic cancer patients during hospitalisation under the background of COVID-19 | Cross-sectional | 100 pancreatic cancer caregivers  55% female  Age ranged from under 30 to over 60  53% spouse, 43% child, 2% parents, 1% sibling, 1% other | Questionnaires: General Information Questionnaire, Family Caregiver Care Burden Scale, HADS, Benefit Discovery Rating Scale, General Self-Efficacy Scale | Main influencing factors of care burden for pancreatic cancer caregivers were economic pressure, anxiety, depression, and self-efficacy |
|  | Xia et al. (2022), USA [70] | Psychosocial wellbeing in the pancreatic cancer patient-caregiver dyad | Cross-sectional, observational | 28 pancreatic cancer caregivers  64.8% female  Mean age 63.6  82% spouse | Questionnaires: The National Comprehensive Cancer Network (NCCN) Distress Thermometer, Perceived Stress Scale 4 (PSS-4), PROMIS-Anxiety, PROMIS-depression, 12-item Caregiver Burden Interview (CBI-12) | Patient distress predicted caregiver distress, anxiety, depression, and perceived burden  Younger caregivers had higher anxiety and caregiver burden  Number of caregiving activities and overall health status of caregiver associated with increased depression and perceived stress |
|  | Yeo et al. (2023), USA [71] | Distress scores in patients with pancreaticobiliary cancers and their significant others | Prospective, descriptive-correlational | 184 significant others  63.5% female (wives, daughters, sisters) | The Distress Thermometer and The Problem List | Significant others reported higher distress scores than patients |
